# A Letter from Brian Berman, President, National Gaucher Foundation

**DOI:** 10.1016/j.ymgmr.2016.10.005

**Published:** 2018-02-12

On June 13, Dr. Roscoe Brady, a scientific legend, passed away, but his legacy lives on in all of us. He had a profound impact on countless people, both through his work and through his kind and caring personality. I owe my life to Dr. Brady and his genius and determination. What follows is my personal account, alongside tributes from some of the others whose lives he improved so dramatically.

In 1983, I was three years old. My spleen was enlarged, my blood counts low and my heart rate abnormally high. I was diagnosed with Gaucher disease, and my prospects were bleak. With no known treatment, the disease progressed quickly.

That was the year I met Dr. Roscoe Brady. Dr. Brady was working under the auspices of the National Institutes of Health (NIH) to develop a treatment for Gaucher disease, and luckily for me, he was ready to test a new enzyme.

My parents worked tirelessly to build support for Dr. Brady's research and to ensure that I could participate in the trial. I would be the first to be successfully treated by the therapy, which is now used to manage Gaucher disease symptoms by thousands of people across the world.

Today, my symptoms are managed and I live a normal, productive life. I also serve as the President of the organization my parents founded all those years ago, the National Gaucher Foundation (NGF). The NGF helps those afflicted by this condition, and we work to educate people about Gaucher disease. NGF also has programs to ensure that everyone who needs treatment for Gaucher disease can access it, regardless of their financial situation.

I was young when he treated me, but I remember Dr. Brady. He was tall and strong. I was scared, and his warmth and gentle hands reassured me. He was charismatic, funny and energetic – but the thing I remember most was his exuberant smile.

It wasn't until much later that I was old enough to understand that Dr. Brady was brilliant – a scientific mind of unparalleled acuteness. His years of hard work and dedication provided me and countless others with a treatment that would give us back our lives.

I have attended the funerals of others who had Gaucher disease – of those who unfortunately developed irreversible complications before any sort of treatment was a viable option. If not for Dr. Brady and his astounding intelligence, his untiring tenacity, and his deep sense of caring and responsibility, that could have been me.

I sit at home late at night, listening to the sounds of my children breathing. If not for the efforts of Dr. Brady, they would not exist. I would not exist.

Dr. Brady certainly had a profound impact on my life and the lives of many others, and for that, we are all immeasurably grateful. In writing this letter about Dr. Brady, I thought it would be fitting to reach out to the leaders of other organizations that represent the countless people around the world who have been so positively affected by his work. Here is what they had to say about this giant of a man. His impact was truly unparalleled.

Tanya Collin-Histed, CEO of the European Gaucher Alliance:

“The EGA greatly mourns the loss, but gives thanks for the life of Roscoe Brady.

Dr. Brady's tireless work at the United States' National Institutes of Health in Bethesda, Maryland to identify metabolic defects in hereditary storage disorders, including Gaucher disease, gave hope and ultimately a treatment to Gaucher patients. By applying his brilliance in developing diagnostic tests, crucially, he was able to develop enzyme replacement therapy as an effective treatment for Gaucher disease and other lysosomal storage disorders.

HIs tenacious approach to pioneering work transformed the future for Gaucher patients; developing a way to replace defective enzymes resulted in a life-saving treatment that has completely changed what would otherwise have been the destiny of those suffering from Gaucher disease. Gaucher patients - and indeed patients of other lysosomal storage disorders -will remain ever grateful to Dr. Brady for the very real difference that he made to their lives.

We have been asked what Roscoe Brady meant to Gaucher patients. The answer is that his work gave so many the chance of living a normal life. Gaucher patients throughout the world and the EGA mourn Dr. Brady's passing, send condolences to his family and give thanks for his life, as his work saved the lives of countless patients across the globe.”

Jerry Walter, Founder and President of the National Fabry Disease Foundation:

“On behalf of the Fabry disease community, we are extremely grateful for the many tremendous contributions of Dr. Roscoe Brady to improving the understanding and management of Fabry disease, and for helping to enable significant improvements to the health, welfare and longevity of people with Fabry disease. One can see that Dr. Brady contributed to Fabry disease research for over 50 years based just on published peer-reviewed journal articles alone, and we know it didn't begin with the first publication we can retrieve from the US National Library of Medicine (PubMed). Dr. Brady spent a lifetime trying to provide people with Fabry disease a chance at better and longer lives.

Personally, it was an honor to know Dr. Brady and to work with him over the years. I first met Dr. Brady in 2002, when approval of the first enzyme replacement therapy for Fabry disease was being considered in the U.S. Later, we corresponded occasionally, and conversed at meetings and while traveling. Once, while sitting across the aisle from each other an airplane several years ago, I learned a great deal about substrate reduction concepts and theories. Dr. Brady always exhibited so much enthusiasm for furthering science on our behalf. We will truly miss this exceptional leader, teacher, explorer, scientist, humanitarian and friend.”

Jack Johnson, Executive Director of Fabry Support & Information Group:

“When I first started patient advocacy work for those with Fabry disease, I didn't know of Dr. Roscoe Brady or how his discoveries laid the foundation for today's improved understandings and treatments for lysosomal storage diseases (LSDs). It was not long, though, until I heard about his work at the National Institutes of Health (NIH). Word of mouth from other patients in the Fabry community spread news of his wonderful work and involvement in a clinical trial to treat Fabry disease.

I first met Dr. Brady at a medical meeting where I got a glimpse of his work and saw the respect others in the medical community held for him. Upon having the opportunity to briefly speak with him, I instantly liked him. Even though I had no medical training, he readily accepted my questions with consideration and answered with a patient manner. He had a talent to make me feel as though my questions or comments were as important as those of his medical colleagues, and he gave the time to bring the high-level science down to the level I could grasp. This, of course, helped me to gain fuller understandings and ultimately to be a better patient advocate.

It was when I and other Fabry-affected family members were evaluated in his program at NIH that I saw what a truly caring man he was. He was always able to put you at ease, but when my aunt became suddenly overwhelmed with all she was facing, Dr. Brady immediately sat on the bed, putting his arm around her shoulders, to calm and help her deal with how she was feeling.

Speaking with Dr. Brady on the patient floor, in his office or at one of the many medical meetings he participated in, he always had a smile, shared a warm laugh and made you feel welcome. It was like he was your welcoming grandfather.

I know Dr. Brady has many well-deserved scientific accolades, but I always think of him as the wonderful man that helped to improve so many lives.”

Peter L. Saltonstall, President and CEO of the National Organization for Rare Disorders:

‘“Dr. Brady was a longtime member of the National Organization for Rare Disorders (NORD) Medical Advisory Committee. In that role, he devoted countless hours to helping NORD provide accurate information on rare metabolic diseases to patients and medical professionals, overseeing NORD's Research Grants Program and advising NORD's advocacy on public policies related to his areas of expertise.

The NORD/Roscoe Brady Lysosomal Storage Disease Fellowships provided significant funding to encourage young researchers to study lysosomal storage diseases. These fellowships resulted in numerous important publications and better understanding of many LSDs.

Beyond those formal affiliations, NORD staff members knew they could contact Dr. Brady at any time and receive a prompt and helpful response, even long after he had “retired” from his laboratory at NIH. In 2010, NORD presented its Lifetime Achievement Award to Dr. Brady and included the following comments in its tribute to him at that time:

“For more than 50 years, Roscoe O. Brady, MD, has conducted pioneering research on hereditary metabolic storage diseases. His work has defined much of what is known today about the biochemistry, enzymatic bases, and metabolic defects of these diseases, and he has inspired colleagues throughout the world to pursue further investigations. NORD is proud to present its own Lifetime Achievement Award to Dr. Brady, and gratefully acknowledges his years of service on the NORD Medical Advisory Committee as a friend and advisor to the rare disease patient community.”’

To everyone who has Gaucher disease, Fabry disease, or knows someone who deals with an LSD, Dr. Brady is a giant of a man. We view him with an awesome sense of appreciation. But, we all agree, immense though his accomplishments are, he should be remembered for much more than his scientific achievements.

He should be remembered for his passion, for his energy, and for his kindness. And, of course, for his smile.

Those of us who were fortunate enough to know Dr. Brady personally know that the world has lost not only a great scientist, but a great man.

Mere appreciation is not enough; Dr. Brady's memory requires action from all of us. He provided us with the gift of life. We must continue his legacy by working as diligently as he did to help those suffering from lysosomal storage diseases. His life and his work is an inspiration for those of us at the National Gaucher Foundation, and I know he is proud of how far we have come.

May we all be as passionate, as energetic, and as kind as Dr. Brady. May we all care as deeply. May we all feel such a sense of responsibility to the world and to each other.

Dr. Brady, may we all live lives that would make you proud, and may your memory forever be a blessing.

